# Preliminary Study of κ-Carrageenan Based Membranes for Anti-Inflammatory Drug Delivery

**DOI:** 10.3390/polym14204275

**Published:** 2022-10-12

**Authors:** Dorinel Okolišan, Gabriela Vlase, Titus Vlase, Claudiu Avram

**Affiliations:** 1 Research Centre for Thermal Analysis in Environmental Problems, West University of Timisoara, Pestalozzi Street 16, 300115 Timisoara, Romania; 2 Physical Therapy and Special Motricity Department, West University of Timisoara, B-Dul V. Parvan No.4, 300223 Timisoara, Romania; 3 Faculty of Medicine, University of Medicine and Pharmacy “Victor Babeş”, Eftimie Murgu Square 2, 300041 Timişoara, Romania

**Keywords:** κ-carrageenan, drug delivery, anti-inflammatory drugs, biopolymers, FT-IR, thermal analisys, UV-Vis

## Abstract

This study proposes a simple and effective method to obtain ultra-thin membranes based on κ-carrageenan. Two types of membranes were obtained, one based on κ-carrageenan and the second type based on κ-carrageenan, hydroxyethyl cellulose and the plasticizer (glycerol). Three non-steroidal anti-inflammatory drugs (Dexketoprofen trometamol, Meloxicam, Diclofenac sodium) and a glucocorticoid (Dexamethasone) were introduced, looking for the best option for incorporation. The obtained membranes were characterized by FTIR, TG/DTG and UV-VIS methods and the data collected following these methods indicated success in terms of the incorporation of the active substance, as well as the high thermal stability in the temperature range 37–100 °C of both the matrices of membrane types, as well as the membranes with the drug incorporated. All the studies carried out led to the conclusion that within all the membranes the anti-inflammatory substances were intact and, thus we can say that these membranes can be used for transdermal administration of the studied anti-inflammatory substances.

## 1. Introduction

Polysaccharides are known for their film-forming properties and are being extensively studied for food and non-food applications. It has been shown that a wide range of film properties can be achieved, due to the variety of polysaccharides available. The use of natural polymer films depends on several properties, including cost, availability, functional properties, mechanical properties (strength and flexibility), optical quality (gloss and opacity), water resistance of the structure, and tactile feel. These properties depend on the type of material used as the structural matrix (morphology, molecular weight, charge distribution), film manufacturing conditions (solvent, pH, concentration, temperature, etc.), type and additive concentration (plasticizer, cross-linking agent, antimicrobial agent, antioxidant, etc.) [1,2].

Biopolymer-based drug delivery systems can improve the pharmacokinetics of drugs, improve therapeutic indices, and reduce side effects to increase the overall system’s effectiveness. Over the years, different types of polymer drug delivery systems have been developed, including microspheres, micelles, hydrogels, nanoparticles, and more. Drug molecules are released from the polymer matrix through various mechanisms, such as diffusion, erosion, polymer degradation, etc. Biodegradable and biocompatible polymers are best suited for this application because adequate drug release is required and because the carrier must be easily removed after drug administration [3].

Polysaccharides have also found application in nano drug delivery systems. Thus, a few years ago, F.A. Oyarzun-Ampuero et al. [4] published a study in which the most well-known natural polysaccharides, chitosan and hyaluronic acid, were used to create a nanosystem to obtain mucoadhesive nanocarriers loaded with heparin, suitable for pulmonary delivery. Polymeric nanoparticles that respond to the surrounding environment have been gaining popularity in the medical field over the past decade. This approach is particularly useful for diagnostic and therapeutic delivery. One representative biomaterial that can be used to design microenvironment-responsive polymeric nanoparticles is hyaluronic acid [5,6].

In recent decades, extensive studies have been conducted on biodegradable films made from various biopolymers based on proteins, polysaccharides, and lipids. κ-carrageenan (KC) is one of the most interesting biopolymers. It consists of a linear chain of sulfated galactans and is extracted from red sea algae [7], particularly from different species of Rhodophyta: Chondrus, Eucheuma, Gigartina, and Hypnea [8,9,10,11,12]. It has high potential for the development of a drug delivery system as it can be used as raw material for biodegradable films.

In multiple animal studies, carrageenan has been linked to the formation and development of intestinal neoplasms and ulceration. The toxicity of this biopolymer is linked to its unusual chemical structure. It can be classified into two types: high molecular weight carrageenan (undegraded) and low molecular weight carrageenan (degraded), with an inflammatory effect [13]. Acidic exposure may cause carrageenan degradation by depolymerization. Degraded k-carrageenan inhibited cell proliferation in Caco-2, FHs 74 Int, HepG2, and Fa2N-4 cell lines, and the anti-proliferative impact was associated with apoptosis and inactivation of cell proliferating genes, as established by morphological and molecular investigation. However, undegraded carrageenan had no cytotoxic impact on normal and malignant intestinal and liver cell lines [14].

Antipyretic, anti-inflammatory, and analgesic properties were identified as the principal therapeutic activities of aspirin (and sodium salicylate itself) by the early 1900s. With time, various additional medications that have some or all of these effects have been found. Antipyrine, phenacetin, acetaminophen (paracetamol), phenylbutazone, and, more recently, thefenamates, indomethacin, and naproxen are among these medications. Due to their comparable therapeutic properties, these medicines were grouped and dubbed as “aspirin-like” medications. These medications were named “nonsteroidal anti-inflammatory drugs”, because they were readily distinguished from glucocorticoids (the other major group of treatments used to treat inflammation) [15,16].

Regardless of their chemical structures, all anti-inflammatory drugs have the same therapeutic qualities. They relieve inflammation swelling, redness, and discomfort, as well as fever and pain. Furthermore, they have several comparable adverse effects, to a greater or lesser extent. They can produce gastrointestinal discomfort, depending on the dose, delay the birth process in high doses, and harm the kidneys in overdoses. The antithrombotic effect is a very interesting side effect, which is now acknowledged as a therapeutic activity [17].

**Figure 1:**
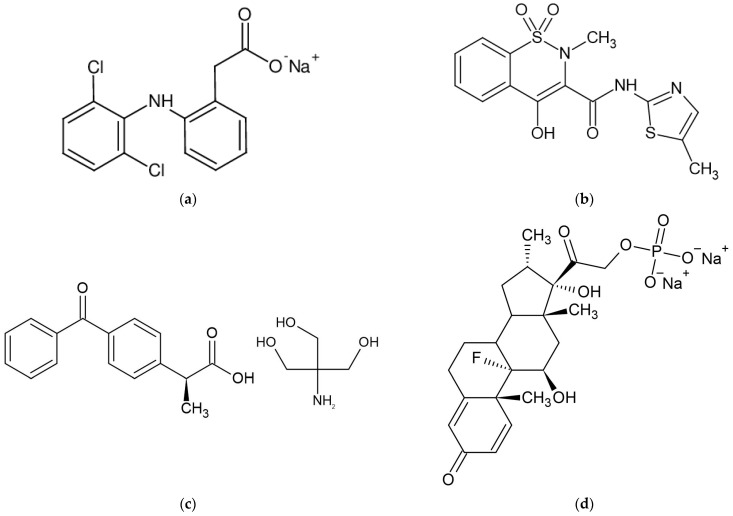
(**a**) Sodium Diclofenac molecule [19]. (**b**) Meloxicam molecule [21]. (**c**) Dexketoprofen trometamol molecule [24]. (**d**) Dexamethasone sodium phosphate molecule [26].

Diclofenac (Ref) (Figure 1a) is an analgesic, anti-inflammatory, and antipyretic nonsteroidal anti-inflammatory drug (NSAID) that has been demonstrated to be useful in the treatment of several acute and chronic pain and inflammatory diseases. Diclofenac, like all NSAIDs, works by inhibiting prostaglandin production by inhibiting both cyclooxygenase-1 (COX-1) and cyclooxygenase-2 (COX-2) with equal efficacy. However, substantial research demonstrates that diclofenac’s pharmacologic activity comprises multimodal and, in some cases, new modes of action in addition to COX inhibition [18,19].

Meloxicam (Mel) (Figure 1b) is a relatively recent NSAID that has been licensed in the United States for the treatment of osteoarthritis. It has also been studied as a therapy for rheumatoid arthritis, ankylosing spondylitis, and acute rheumatic pain. Meloxicam has been demonstrated to be COX-2 selective, especially at the lowest therapeutic dosage, and to have anti-inflammatory properties by decreasing prostanoid production in inflammatory cells. It is believed to have less GI toxicity than nonselective NSAIDs since it is COX-2 preferential. Meloxicam was shown to be equally effective as piroxicam, diclofenac, and naproxen in clinical studies for osteoarthritis, with fewer GI symptoms and a decreased incidence of ulceration, and bleeding [20,21].

Dexketoprofen (Tad) (Figure 1c) is a non-steroidal anti-inflammatory drug that is derived from aryl propionic acid. Ketoprofen is an anti-inflammatory, analgesic, and antipyretic medication with a long history of use. Dexketoprofen is the S-form of ketoprofen and is more potent than the racemic mixture. The dexketoprofen trometamol, the salt form of dexketoprofen, is absorbed fast and gives a patient a high blood level of the medication. This can be beneficial for patients with pain in the moderate to severe range, and has been shown to give good pain relief with a quick onset of action. Dexketoprofen was created to shorten the time it takes for the painkilling effect, to increase the potency of the drug, and to reduce gastrointestinal side effects [22,23,24].

As part of the synthetic glucocorticoid class, Dexamethasone (Dex) (Figure 1d) is used as an anti-inflammatory and analgesic drug, but, more recently, during the COVID-19 pandemic it was used as an adjunct treatment that helped lower the mortality rate among patients with a severe form of SARS-CoV-2 infection [25].

Dexamethasone sodium phosphate or pregnant-1,4-diene-3,20-dione,9-fluoro-11,17-dihydroxy-16-methyl-21-(phosphonoooxy)-disodium salt, is dexamethasone’s inorganic ester that is widely used in chemotherapy patients and to treat various dermatologic, endocrinologic, rheumatologic and allergic problems [27].

The present study aimed to obtain the best pharmaceutical formula with a carrageenan-based membrane, which contained various active substances with an anti-inflammatory role, for possible transdermal application to eliminate the side effects highlighted in the case of oral administration.

The presented study managed to establish, by combining the results of several complementary physico-chemical techniques (TG, FTIR, UV-Vis) [28,29], which would be the best membrane variant that could be used to release the studied active substances.

## 2. Materials and Methods

### 2.1. Chemicals

Biopolymers used for membrane preparation were κ-carrageenan by Acros Organics (Geel, Belgian), with a molecular weight of 788.647 g/mol, CAS number: 11114-20-8, and hydroxyethyl cellulose sold by Sigma Aldrich, CAS number: 9004-62-0. The plasticizer used for these membranes was glycerin, sold by CHIMREACTIV (Ion Creanga, Romania), with a molecular weight of 92.10 g/mol, CAS number: 56-81-5.

Anti-inflammatory drugs, as injectable solutions, used in this study were: Sodium diclofenac (25 mg/mL), marketed as Refen™ by STADA HEMOFARM (Vršac, Serbia), Dexketoprofen trometamol (25 mg/mL), commercialized as Tador™ by MENARINI I (Florence, Italy), Meloxicam (10 mg/mL), produced by Rompharm (Otopeni, Romania) and Dexamethasone phosphate (4 mg/mL), marketed as Dexametazonă and also produced by Rompfarm.

### 2.2. Methods

**Biopolymer membrane synthesis**

To prepare biopolymer membranes, different combinations and mass ratios between biopolymer and plasticizer were tested, using protocols adapted from Rukmanikrishnan et al [30], originally used for the production of ultra-thin packaging films. Of these, two types of membranes were chosen, which were considered to be the best for the incorporation of anti-inflammatory drugs.

**The first type of membrane (A)** was prepared by mixing glycerol and κ-carrageenan (in a 0.6:1 ration, *w*/*w*) and dissolving the mixture in distilled water in a Berzelius beaker on a magnetic stirrer at 500 rpm until the entire amount of biopolymer was completely solubilized to obtain a stock solution. To prepare a biopolymer membrane, a required volume of this stock solution was transferred, using an automatic pipette, into a suitable Berzelius beaker and a calculated volume of the drug was added and stirred on a hot plate (volume of the active drug was calculated respecting the ratio 4:1 *w*/*w*). The obtained solutions were poured into small Petri dishes and left to air dry. After complete drying, the membranes were analyzed.

**The second type of membrane** (**B**) was prepared following this recipe: into a suitable Berzelius beaker, weighed amounts of glycerol (as 75% of dry components), κ-carrageenan, and hydroxyethyl cellulose (in a 1:1 ration, *w*/*w*) were placed and 10 mL of distilled water were added. The beaker was placed on a magnetic stirrer until a homogenous stock solution was obtained. For membrane preparation, the calculated volume of this stock solution and drug were added into a proper beaker and stirred on a hot plate (the mass ratio of 4:1 was respected, as mentioned above). The obtained solution was poured into a Petri dish and, following the drying steps described above, we obtained an ultra-thin biopolymer membrane.

**Synthesis of membranes with active substances**: The ratio was 4:1 (components in solid form: active substance). In the case of type A membranes, 80 mg (membrane base) and 20 mg of active substance, and for type B, 133 mg (membrane base) and 33.25 mg of active substance.

FTIR analysis

Data collection was performed after 20 recordings at a resolution of 4 cm^−1^, in the range of 4000–400 cm^−1^ on a Shimadzu FT-IR Spectrometer IRTracer-100 with ATR.

TG/DTG analysis

Samples analysis was performed in an air atmosphere (50 mL∙min^−1^), in a temperature range of 25–500 °C, with a heating rate of 10 °C∙min^−1^ on an METTLER TOLEDO Thermogravimetric Analyzer, model TGA/DSC^3+^ in open aluminum crucibles.

UV-Vis Spectrophotometry

Samples were analyzed using an Agilent UV-Vis Spectrophotometer, model Cary 60, in the range of 200–600 nm and with a UV-Vis scan rate of 600 nm·min^−1^ and UV-Vis data interval of 1.00 nm.

## 3. Results and Discussion

### 3.1. Membrane Formulation

Following the work steps described above, two types of membranes were obtained: Type A membranes (CG) and type B membranes (CCG). Due to the different mass ratios between biopolymers and plasticizers, these two types of membranes had different aspects. The first type of membranes had an elasticity similar to that of cellophane and although they were thin they were not brittle. The second type of membranes, in which, in addition to carrageenan and glycerin, hydroxyethyl cellulose (added in a mass ratio of 1:1 to carrageenan) was also present, were like a stretch film and had a higher elasticity than type A membranes. In addition, due to the higher amount of glycerin, the second type of membranes could adhere very well to the skin, which could be an advantage if intended for transdermal drug delivery. It should be noted that both types of membranes were obtained as very thin films.

Although in the literature, the membranes were dried in the oven and at various temperatures, that were usually higher than the ambient temperature, the membranes we obtained dried successfully at room temperature. This process was slower compared to the literature, taking 48 h for type A membranes and 72 h for type B membranes.

Table 1 shows the membranes in which four types of anti-inflammatory drugs were incorporated. Of these four, three were nonsteroidal anti-inflammatory drugs (Ref, Tad and Mel) and the only steroidal drug was Dex. First of all, we had a single- colored membrane, CGMel, where the staining was due to the Mel solution which had a straw-yellow color. Although colored, it did not show traces of the crystallized substance, and the membrane was relatively transparent. The CGTad membrane was the only completely transparent membrane that had a smooth surface, which could be deduced from the reflection of light on its surface. The only membranes in which crystallization of the active substance could be observed were CGDex and CGRef. From the appearance of the CGDex membrane we can see that, although the active substance had relatively large molecules, it was fully incorporated into the membrane. Unlike the CGRef membrane, which was transparent and in which we could see that the drug had crystallized, with small conglomerations distributed throughout the membrane, the CGDex membrane was opaque. Although the appearance of these membranes varied, they all had mechanical strength.

Membranes prepared according to recipe B, in which anti-inflammatory drugs were incorporated are shown in Table 1. As for the drugs that were incorporated, they behaved exactly as in the case of type A membranes. Namely, Meloxicam contributed to the color of the membrane (CCGMel), and sodium diclofenac (CCGRef) crystallized, as did Dexamethasone (CCGDex). An interesting thing to note is that although the CCGDex membrane was elastic, as were the other type B membranes, it was, at the same time, more fragile, and broke very easily. Table 1 is presented together with the pictures of the membranes and the images obtained with the Shimadzu IR-AIM 9000 Microscope was visible by reflection with a scale of 500 µm, which showed us that in all situations the membranes obtained were homogeneous.

### 3.2. FT-IR Spectra

**Figure 2:**
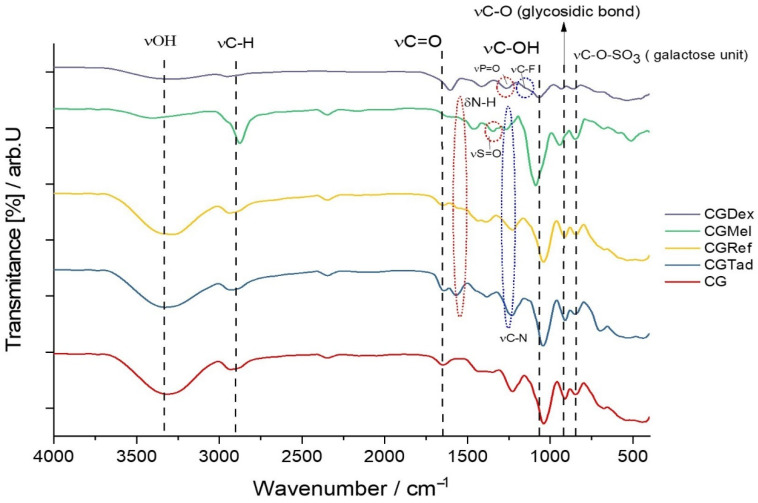
FTIR spectra for the following membranes CG, CGDex, CGMel, CGref, and CGTad.

Figure 2 shows the FTIR spectrum for the CG membrane, a type-A membrane, in which no active substance was incorporated. Given that the membrane consists of only a biopolymer and plasticizer, a simple binary mixture, the signals obtained from the FTIR analysis corresponded to the following bond vibrations: the wide signal extended over the region of 3100–3600 cm^−1^ (the highest absorption being at 3315.63 cm^−1^ in this case) and was attributed to O-H bond vibration, due to the presence of the hydroxyl group in both glycerin and κ-carrageenan. The absorption bands at 2937.59 cm^−1^ and 2885.51 cm^−1^ corresponded to the stretching vibration of the C-H bond, characteristic of the aliphatic chain in which carbon has a sp^3^ hybridization. It was expected that these two types of signals would occur because κ-carrageenan, being a polysaccharide, and glycerin-trihydric alcohol, are plentifully loaded with hydroxyl groups and sp^3^ hybridized carbon, hence a great number of C-H and O-H bonds were present.

Since κ-carrageenan is composed of repetitive units of galactose, an aldohexose, the signal showing the maximum absorption at 1647.21 cm^−1^ was characteristic of the aldehyde carbonyl that was present in this compound. Knowing that galactose is sulfonated, the characteristic peak of the stretching vibration of the S=O bond showed an absorption maximum at 1220.94 cm^−1^. The most intense peaks, at 1031.46 cm^−1^ and 918.63 cm^−1^, corresponded to the C-O stretching vibration, both from the C-OH and C-O glycosidic bond (linkage of 3,6-anhydro-D-galactose units). The absorption signal from 1419.61 cm^−1^ and 1367.53 cm^−1^ was due to the bending vibration of C-OH, and the one from 1161.15 cm^−1^ represented the symmetrical deformations of the C-H bond. Although the fingerprint region was sometimes exceedingly difficult to interpret, according to data from the literature, as can be seen in figure, the κ-carrageenan had a unique “fingerprint”, located, in this case, at 844.82 cm^−1^, which could be seen in almost all the membranes made. This absorption maximum showed the presence of C-O-SO_3_ of D-galactose-4, sulfate.

As mentioned earlier, in type A membranes different anti-inflammatory drugs were incorporated and, therefore, the obtained membranes were subjected to FTIR analysis. Thus, the resulting spectra were compared with the control membrane and plotted graphically (Figure 2). 

Wavenumber values of the most relevant peaks for each membrane containing anti-inflammatory drugs are shown in Table 2.

Since the matrices of all membranes were composed of κ-carrageenan and glycerin, the characteristic peaks of these two compounds could be found in all the spectra represented in Figure 2. At first glance at the figure, we can see that around 1000–1050 cm^−1^ was the most intense peak that corresponded to C-O stretching, and linkage of 3,6-anhydro-D-galactose. Only in the case of the CGDex membrane, the peak of 1037.70 had a much lower percentage of transmission, which could be the result of the following: the pH of the injectable dexamethasone solution was between 7–8.5, a slightly alkaline pH, which could affect the integrity of the polymer, i.e., k-carrageenan. Therefore, this pH could induce slight depolymerization of carrageenan, by breaking glycosidic bonds (-C-O-C-). As is known, the higher the number of bonds of a certain type, in this case the -C-O-C- bond, the higher the intensity of the peak that this bond has in the IR spectrum. We can say that in the case of the CGDex membrane, although it was obtained successfully, the peak intensity was lower and a change in the -C-O-C- bond had occurred.

Characteristic peaks of the control membrane that could be found in membranes with anti-inflammatory drugs were as follows: stretching vibration of the O-H bond (3100–3400 cm^−1^), stretching vibration of the C-H bond (2800–2900 cm^−1^), stretching vibration of the aldehyde carbonyl of galactose (1640–1700 cm^−1^), C-O bond stretching from C-O-C linkage of 3,6-anhydro-D-galactose (two peaks, 1030–1060 cm^−1^; 918–950 cm^−1^) and vibration for C-O-SO3 of D-galactose-4, sulfates (840–844 cm^−1^). A particular feature associated with the spectrum of the CGMel membrane was the intense peak at 2879.72 cm^−1^. Although naturally the Meloxicam molecule had C-H bonds, the peak of this wave number was given to a greater extent to the R2N-CH3 vibration, more precisely to the C-H bond vibration of the methyl radical, associated with the nitrogen atom entering the molecular structure of Meloxicam. The vibration at 1462.04 cm^−1^ was due to = C-H and ring C=C structural vibration of the benzene moiety of Meloxicam and the one at 1338.04 cm^−1^ represented the S=O stretch from the sulfone group (R-(O=S=O)-R) in 4-hydroxy-2-methyl-5,6-dihydro-1λ^6^,2-thiazine-1,1(2H)-dione moiety of Mel.

In the case of the CGDex membrane, in addition to the peaks representative of the control membrane, some characteristic peaks could be seen, which could be attributed to important constituent parts of the dexamethasone phosphate molecule, namely the following: stretch vibrations of C=C at 1579.70 cm^−1^ from 2,5-cyclohexadien-1-one, R-CH_3_ bond stretch vibrations at 1390.68 cm^−^^1^, stretch vibrations of P=O bond at 1240.23 cm^−1^ and stretch vibrations of C-F bond at 1155.36 cm^−1^. In addition to the peaks associated with the control membrane, the CGRef and CGTad membranes had characteristic peaks for the vibration of the C=C bond in aromatics. The first membrane had a peak at 1379.10 cm^−1^ and the second one at 1245 cm^−1^. In addition, CGRef also showed a peak in the fingerprint region at 700.16 cm^−1^ for the stretch vibration of the C-Cl bond.

**Figure 3:**
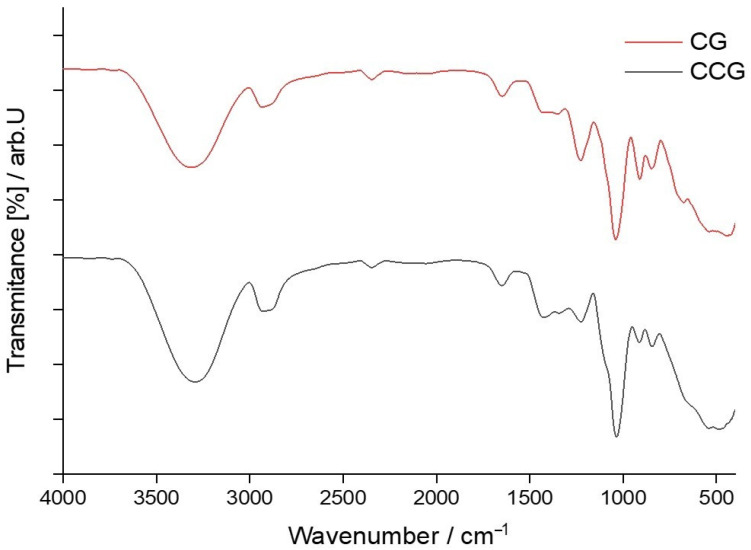
Superposed FTIR spectra of control membranes, CG and CCG.

Unlike type A membranes, type B membranes were composed of a mixture of three components, k-carrageenan, glycerol and hydroxyethyl cellulose, and, thus, the need for FTIR analysis of type B control membrane was more than necessary. To compare more easily the effect that the addition of hydroxyethyl cellulose had on the intensity of peaks that appeared in the FTIR spectrum of type B control membranes, both type A and type B spectra, for control membranes, were graphically plotted and are shown in Figure 3. 

Knowing that the composition of the membranes differed, it was clear that their FTIR spectrum would also vary. The amount of κ-carrageenan, glycerin and hydroxyethyl cellulose directly influenced the intensity of the peaks. Therefore, the fact that the amount of κ-carrageenan that entered the composition of type B membranes was much smaller, compared to type A membranes, could be deduced from the intensity of the characteristic peaks for this biopolymer, namely:

1. An obvious decrease in peak intensity for C-O-SO_3_ vibration of C4 of galactose at 86,7848.68 cm^−1^ and with a transmittance of 62.65% was visible for the CCG membrane, since the transmittance for the same type of vibration found at 844.22 cm^−1^ was 53.86% in the case of CG membrane.
2. There was a decrease in peak intensity corresponding to C-O bond stretching, and linkage of 3,6-anhydro-D-galactose. In the case of the CCG membrane, the stretching vibration from 920.05 cm ^−1^ had a transmittance of 59.83% while the same vibration at 918.12 cm^−1^ in the case of the CG membrane had a transmittance of 50.22%. Although this decrease in the percentage of transmitted light was not so intense, since some of the IR light was also absorbed by the C-O-C glycosidic bonds between the glucose units in hydroxyethyl cellulose.
3. A decrease in peak intensity for S=O stretching vibration was also observable. For the CCG membrane this peak at 1219.01 cm^−1^ had a transmittance of 75.63% while for the CG membrane the transmittance for the same vibration at 1220.94 cm^−1^ was 64.79%.

A closer look at the superposed spectra of membranes in Figure 3, shows that there was a significant increase in the intensity of the peaks corresponding to the stretching vibrations of the C-H and O-H bonds in the CCG membrane. These increases in transmittance were due to the introduction of hydroxyethyl cellulose into the membrane matrix. Hydroxyethyl cellulose is a polysaccharide and is, therefore, rich in hydroxyl groups and sp^3^ hybridized carbon contributes in a significant way to the overall increase of the transmittance for the peaks that corresponded to the stretching vibrations of the bonds in question. In the case of the O-H bond vibration, which, in the CG membrane, had a transmittance of 84.19% at 2937.59 cm^−1^, had, in the CCG membrane, a transmittance of 78.44% at 2935.66 cm^−1^. The decrease in transmittance indicated an increase in absorbance which practically meant that a larger number of bonds that had a maximum of absorption at these values of the wavenumbers were present in the membrane matrix.

**Figure 4:**
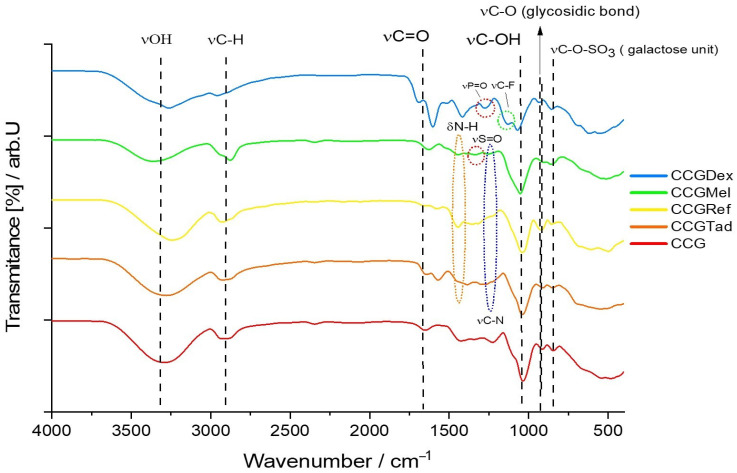
FTIR spectra for the following membranes CCG, CCGDex, CCGMel, CCGref, CCGTad.

For type B membranes, in which the four anti-inflammatory drugs were incorporated, the FTIR spectrum is shown in Figure 4. The spectrum of membranes with the active substances was compared graphically with the control membrane, so that eventual changes in the membrane matrix, caused by the active substances themselves, could be easier to spot.

As in the case of the CGDex membrane, both the characteristic peaks of the dexamethasone phosphate and the membrane matrix were present in the FTIR spectrum of the CCGDex membrane. Thus, at a wave number of 1579.70 cm-^1^ and transmittance of 39.83% there was a peak for the vibration of the C=C bond, and at 1390.68 cm^−1^ and transmittance of 50.29% there was the characteristic peak for symmetrical deformation of the R-CH_3_ bond. This peak of the C=C bond was due to dexamethasone itself, more precisely the 2,5-cyclohexadien-1-one moiety, and the peak for R-CH3 vibration was due to the methyl radicals found on both the dexamethasone and hydroxyethyl cellulose molecules. As for the rest of the membranes, CCGMel, CCGRef, CCGTad, their characteristic peaks for some functional groups were visible at wavenumbers close to those of the CG membranes, but with a different percentage of transmittance, being more or less intense. 

The wavenumber values of the most relevant peaks found in the FTIR spectra of all type B membranes are shown in Table 3.

### 3.3. TG/DTG Analysis

**Figure 5:**
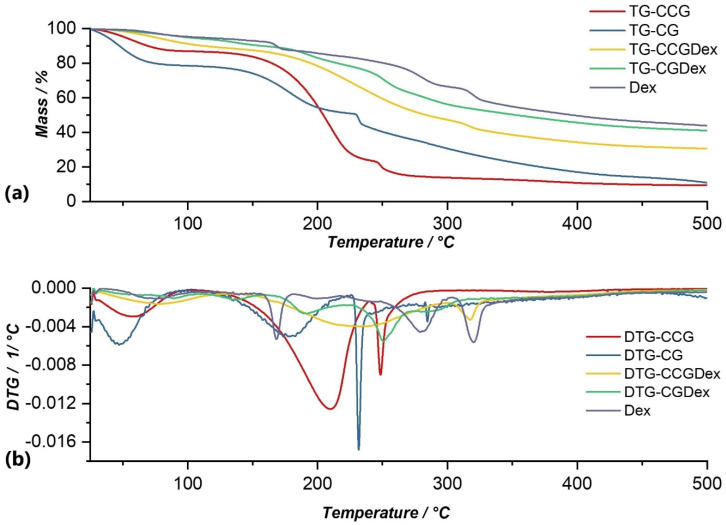
Comparison of TG (**a**) and DTG (**b**) curves of Dex with membranes in which this substance was incorporated (CCGDex, CGDex), as well as with control membranes (CCG and CG).

**Figure 6:**
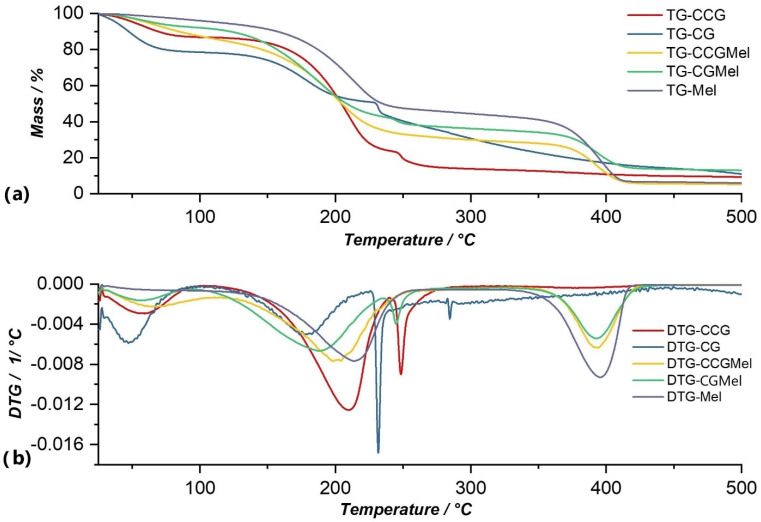
Comparison of TG (**a**) and DTG (**b**) curves of Mel with membranes in which this substance was incorporated (CCGMel, CGMel), as well as with control membranes (CCG and CG).

**Figure 7:**
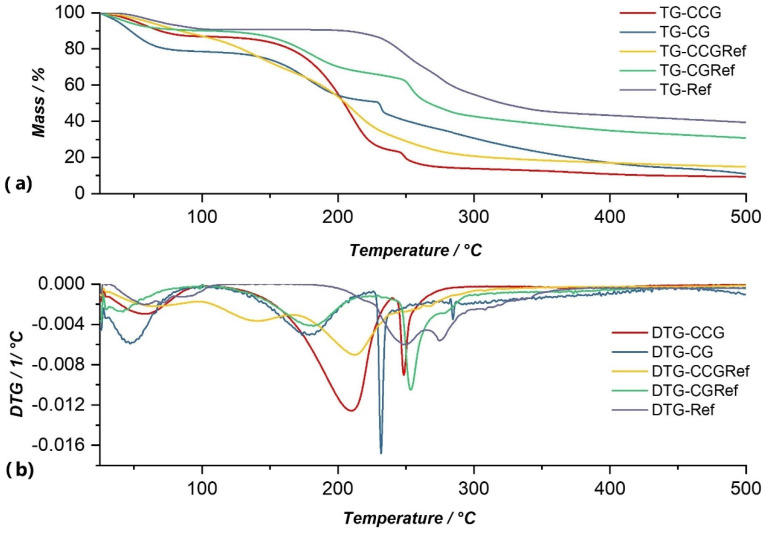
Comparison of TG (**a**) and DTG (**b**) curves of Ref with membranes in which this substance was incorporated (CCGRef, CGRef), as well as with control membranes (CCG and CG).

**Figure 8:**
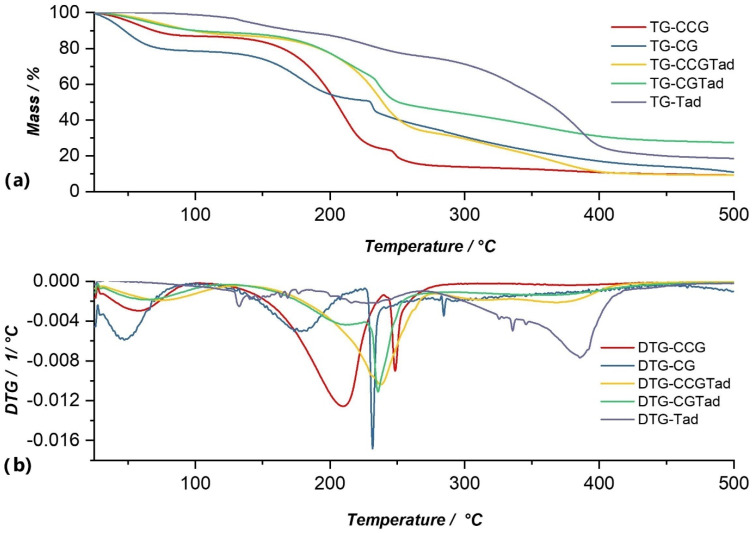
Comparison of TG (**a**) and DTG (**b**) curves of Tad with membranes in which this substance was incorporated (CCGTad, CGTad), as well as with control membranes (CCG and CG).

Data obtained from thermogravimetric analysis for anti-inflammatory drugs that were used in this study are graphically represented in their TG and DTG curves in Figure 5, Figure 6, Figure 7 and Figure 8, and are compared with both type A and type B membranes in which anti-inflammatory drugs were incorporated, as well as with the control membranes (CG and CCG). 

Thermal analysis of Dexamethasone revealed 5 processes of thermal decomposition (Figure 5). The first process that can be observed on the DTG curve (Figure 5b) could be attributed to crystallization water losses. Following this process, the mass loss was 4.15% of the initial mass of the sample. This process was followed by four processes in which the thermal decomposition of dexamethasone occurred. Of these, the most significant were the third and fourth processes, in which the mass losses were 20.27%, and 12.46% respectively, in the temperature ranges of 219.90–309.16 °C and 309.16–361.44 °C, respectively. Thus, in these two processes, the complete disintegration of dexamethasone took place so that the total mass loss was 45.71%, in the temperature range of 39.74–361.44 °C.

The thermal decomposition of meloxicam occurred through two intense processes that can be observed in the DTG curve (Figure 6b). Thus, the first process occurred at a temperature range of 109.72–265.53 °C and resulted in a loss of 53.13% of the initial mass of the sample. This process was due to intermolecular water loss but could also be attributed to the beginning of degradation of the active substance, because, as in the case of the membrane matrix, around temperatures of 200–220 °C there was a rupture of C-S bonds and the formation of sulfur dioxide. The second decomposition process occurred in the temperature range of 323.63–443.44 °C with a mass loss of 32.70%, and was due to proper decomposition of the meloxicam molecule. This process clearly resulted in meloxicam decompositions because these could be observed in the cases of membranes in which this substance was entrapped (CGMel and CCGMel). The total loss from this analysis was 85.14%, from the sample mass.

In the case of sodium diclofenac (Figure 7), the thermal decomposition took place in the temperature range of 38.18–353.39 °C and with a total loss of 2.30 mg, and occurred through three decomposition processes. The first process was due to moisture loss and occurred at a temperature of 38.18–82.52 °C, with a total loss of 9.66% of the initial mass of the sample. There was no clear boundary between the second and third decomposition processes, so they could be considered as a single process. In the latter, the thermal decomposition of the active substance occurred in the temperature range of 220.20–353.39 °C and with a mass loss of 52.79%. Following this thermal analysis, the total loss was 62.46%, in the temperature range of 38.18 −353.39 °C.

The thermal decomposition of Dexketoprofen trometamol proceeded through three decomposition processes, the first of which was a complex process, a multiplex (Figure 8). In this multiplex, the weight loss was 8.67%. The second process occurred at a temperature range of 183.76–266.59 °C and with a mass loss of 12.85%. The highest mass loss was 55.76% and occurred in the third process at a temperature range of 273.04–375.10 °C. These two processes were attributed to the thermal decomposition of the active substance and could be observed in the cases of membranes in which the anti-inflammatory was incorporated (CGTad and CCGTad), but with a lower intensity because the amount was lower, compared to the pure substance.

From the graphical representation of the TG and DTG curves for type A membranes (Figure 5, Figure 6, Figure 7 and Figure 8.) we concluded the following:

1. Of all type A membranes, the CGDex membrane had the lowest percentage of mass loss, namely 58.87%. The first decomposition process of this membrane began at 82.96 °C, unlike the other membranes, whose first processes of moisture removal began in the temperature range of 37–46 °C. A possible explanation could be the fact that the CGDex membrane, in the drying process at room temperature, dried more than the other membranes because it was more fragile after 24 h of drying. Dexamethasone might prevent, to some degree, water from binding to the polymer matrix, which might explain why it did not have a moisture removal process and why the process of water elimination from the polymer matrix was the first process that can be observed on the DTG curve.
2. In the DTG curve of all analyzed membranes, the process of removing water from the membrane matrix was followed by the decomposition of the membrane; more precisely, the decomposition of the polymer found in the membrane. The process of membrane decomposition was intertwined with the process of active substance decomposition. This meant that, once the membrane had decomposed, the decomposition of the active substance began, so it was practically a continuous decomposition which, in the case of membranes such as CGRef, CGDex, and CGTad, could not be delimited.
3. The CGMel membrane was unique because it had a separate decomposition process for the active substance. Most active substances had a decomposition process that started around temperatures of 220–230 °C. In the case of the CGMel membrane, the decomposition process of Meloxicam was in the temperature range of 345.51–442.11 °C.

The thermal analysis of the type A membrane, control membrane CG, revealed four decomposition processes, of which three had a significant percentage of lost mass and one, the final one, smaller mass loss (Figure 6). The percentages of mass loss were in the temperature range of 32.65–292.53 °C. The mass loss of 16.19% after the first process, which occurred in the temperature range of 32.63–67.44 °C, corresponded to moisture loss. At a temperature range of 127.97–209.77 °C, the second process that can be observed on the DTG curve, in which there was a mass loss of 24.25% of the sample mass, was the elimination of water incorporated in the membrane matrix. The degradation of the polymer began in the third process at a temperature range of 222.49–248.99 °C, with a mass loss of 14.56% of the sample mass. In the process, -OSO^3−^ broke off the polymer backbone. Finally, a relatively small process could be observed at a temperature range of 278.74–292.53 °C, in which the glycosidic bonds between the monomer units of κ-carrageenan were broken by a mass loss of 2.01%. Following the TG analysis of the CG membrane, there was a total decomposition of 89.03% [31].

For the CGMel membrane, the thermal analysis revealed three thermal processes that occurred at a temperature range of 37.98–442.11 °C (Figure 6). The first process in which there was a mass loss of 7.16%, with a maximum at 58.45 °C, was due to moisture loss. The second process, which was practically composed of two processes, occurred in the temperature range of 106.90–232.77 °C at a maximum of 172.77 °C. This corresponded to water being eliminated from the membrane and to membrane degradation, with a mass loss of 48.58% of the total sample mass. In the fourth and last process the degradation of the active substance, Meloxicam, occurred in a temperature range of 345.51–442.11 °C. This was characterized by a maximum at 422.85 °C and a mass loss of 21.19%. The CGMel membrane, following this TG analysis, had a total loss of 87.00% of the mass of the analyzed sample.

The thermal decomposition of the CGDex membrane (Figure 5) occurred in a temperature range of 82.96–270.53 °C through four processes, following which there was an observed mass loss of 58.87%. Following the first two decomposition processes, which resulted in a mass loss of 2.24% and 3.34%, respectively, the elimination of water occurred. In the third process, at a temperature range of 178.38–201.09 °C, the degradation of the membrane began with the decomposition of the polysaccharide, resulting in a mass loss of 10.54%. The last process that could be observed on the DTG curve, in the temperature range of 241.99–270.53 °C, corresponded to the thermal degradation of the active substance, Dexamethasone phosphate, with a loss of 23.47%, and a maximum temperature of 250.54 °C. Thus, the decomposition process of the CGDex membrane resulted in a total loss of 58.87% of the total mass of the sample under analysis.

Through the thermal analysis of the CGRef membrane, the DTG curve highlights three processes, as a result of which the total mass loss was 69.17% (Figure 7b). Some of the water present in the membrane was removed in the first process, which can be observed on the DTG curve, and which resulted in a mass loss of 6.66%, followed by the removal of water from the membrane matrix in the second process at a temperature range of 138.96–221.67 °C. In the third process, observable on the DTG curve, the complete decomposition of the active substance occurred, with a mass loss of 22.95%. Thus, following the thermogravimetric analysis to which this membrane was subjected, the mass loss was 69.17% of the entire amount of sample subjected to the analysis.

The DTG curve of the CGTad membrane shows three processes of thermal decomposition, which resulted in a total cumulative mass loss of 72.56% (Figure 8b). As with the other membranes, the first process on the DTG curve of the CGTad membrane was associated with water loss. This process resulted in a mass loss of 10.40%, at a maximum of 67.38 °C. The second process, with a mass loss of 43.64%, corresponded to membrane degradation. The third process, which had a loss of 14.66%, was due to decomposition of the active substance in the temperature range of 329.53–405.86 °C. Following the thermal decomposition processes of the CGTad membrane, the total mass loss was 72.56% of the total mass of the sample under analysis.

For type B membranes in which anti-inflammatory drugs were incorporated, the TG and DTG curves are shown in Figure 5, Figure 6, Figure 7 and Figure 8. The curves of these membranes were compared with the TG and DTG curves for the control membrane, the CCG membrane.

From the graphical representation of the TG and DTG curves for the type B membranes in which the anti-inflammatory drugs were incorporated, we could deduce the following:

1. For the CCG membrane, the decomposition process of the matrix was much more pronounced, because the addition of another biopolymer, hydroxyethyl cellulose- polysaccharide, directly contributed to the increase in the intensity of this process. Basically, by adding HEC, the number of glycosidic bonds that had to be broken increased, and, therefore, the amount of thermal energy that had to be brought to the system for these bonds to break increased. This directly led to an increase in the temperature range in which the thermal decomposition of the CCG membrane matrix occurred.
2. On the DTG curve of the CCGDex membrane we can observe a shift in the decomposition process of the active substance. Thus, compared to the CGDex membrane with a temperature range between 241.99–270.53 °C, in the case of the CCGDex membrane, the process was located in the temperature range of 304.23–335.56 °C. Through a closer analysis of the maximum peak values for all the analyzed membranes, and presented in the tables above, a clear shift in the process corresponding to the decomposition of the active substance could be observed.
3. As in the case of the CGMel membrane, the process corresponding to the thermal decomposition of Meloxicam in the CCGMel membrane was very pronounced and easily observable. This intensity was due to the amount of energy that had to be brought in to decompose the entire amount of meloxicam.

From the graphical representation of the TG and DTG graphs for the type B membrane, CCG control membrane, (Figure 5, Figure 6, Figure 7 and Figure 8) three processes can be observed, but, more precisely, we can say that there were two processes, because no observable limit could be distinguished between the second and third processes. Following the first process, there was a mass loss of 12.12%, which occurred in the temperature range of 36.38–99.94 °C, and corresponded to moisture loss. At a temperature range of 138.89–235.00 °C, the second process could be observed, in which there was a mass loss of 62.79% of the sample mass, due to the elimination of water incorporated in the membrane matrix, but also due to the degradation of the polymer matrix (CG, HEC), which also began and which continued in the third process at a temperature range of 242.27–290.09 °C, with a mass loss of 9.80% of the sample mass. Following the TG analysis of the CCG membrane, there was a total observed loss of 90.63%.

In the case of the CCGDex membrane (Figure 5), thermal decomposition occurred through three processes. In the temperature range of 37.18–129.47 °C, moisture was eliminated with a mass loss. This process had a maximum of 74.40 °C. The removal of water from the membrane matrix and the degradation of the polymer matrix occurred in the second process, at a temperature range of 136.57–301.59 °C. This process had the highest weight loss, namely 40.02%. The third process of thermal degradation of the active substance, Dexamethasone phosphate, occurred in the temperature range of 304.23–335.56 °C, with a loss of 6.62% of the total mass of the sample subjected to analysis. This process was followed by a series of small processes, a complex process of 6 inseparable stages, which were attributed to the breaking of the C-F and C-OPO_3_H bonds of the active substance and which could explain their appearance on the DTG curve.

The DTG curve for the CCGRef membrane highlights three processes of thermal decomposition of the membrane (Figure 7b). If we analyze the DTG curve of the membrane, we can see that the processes were not so distinct, rather proceeding continuously. Loss of humidity occurred in the first process and in the second one loss of water from the membrane matrix and the beginning of the degradation of the polymers occurred. The first process occurred in the temperature range of 35.73–96.51 °C and the second in the temperature range of 130.60–164.86 °C. In the case of the first one, the weight loss was 11.93%, and for the second one, the loss was 20.50%, of the total mass of the sample. In the third process, the degradation of the membrane continued, but at about 259 °C the degradation of the active substance began (as in the case of the CGRef membrane). In this last process the mass loss was 45.26% of the total mass of the sample, a percentage that far exceeded the loss resulting from the first two processes. The total weight loss as a result of these processes was 85.08% of the sample mass.

Thermal analysis of the CCGMel membrane highlights three distinctive decomposition processes that can be seen on the DTG curve of this membrane (Figure 6b). The first process could be linked to a loss of moisture that occurred at a temperature range of 37.83–114.77 °C and with a mass loss of 14.71%. In the second process, which had a loss of 52.33% of the total mass of the sample, the removal of water from the membrane and the degradation of the constituent polymers occurred. The thermal decomposition of the active substance occurred in the third process, in which the loss was 22.88% of the total mass of the sample, in the temperature range of 341.26–454.99 °C. The decomposition process of the active substance in the CCGMel membrane was located in almost the same temperature range as in the case of the CGMel membrane. The total loss from this thermogravimetric analysis was 94.69% of the initial mass of the sample.

Thermal decomposition of the CCGTad membrane proceeded through three processes that can be observed on its DTG curve (Figure 8b). The first decomposition process, with a loss of 12.22%, was attributed to the loss of membrane moisture. In the second process, the degradation of the membrane matrix began, and also the elimination of water continued, with a total loss of 54.25% of the total mass of the sample. The third process was attributed to the decomposition of the active substance, because, as in the case of the CGTad membrane, the active substance began to decompose at a temperature higher than 320 °C. The third process had a total loss of 21.73% of the sample mass, with a maximum of 393.63 °C. Following thermal analysis of the CCGTad membrane, its decomposition occurred in the temperature range of 37.61–426.77 °C, with a total mass loss of 90.82%.

From thermal analysis of type A membranes with anti-inflammatory substances it can be said that CGRef, CGMel and CGDex membranes clearly showed the degradation processes of anti-inflammatory substances, but with maxima on the DTG curves at lower temperatures, which was supported by the fact that the active substances were dispersed in membranes. In the case of the CGTad membrane, the breakdown of the active substance overlapped with the breakdown of the membrane. The same conclusions could be drawn for the B-type membrane.

### 3.4. UV-Vis Spectrophotometry

The spectrophotometric analysis that was applied to the samples aimed to determine the presence or absence of active substances in the membranes. Thus, both type A and type B membranes were subjected to this analysis. In order to confirm, by the UV-Vis method, the presence of active substances in the obtained membranes, samples of membranes and active substances (control solutions) were prepared as follows:

For type A membranes, 200 mg of dry membrane (CGDex, CGMel, CGRef and CGTad) were weighed and dissolved in 1 mL of distilled water. Knowing that 20 mg of active substance was present in each membrane, through calculations it was found that each sample, weighed and dissolved, resulted in a solution of 0.81 mg of active substance/mL. Therefore, control solutions of the active substances were prepared so as to obtain the same concentration.

For type B membranes, 100 mg of dry membrane (CCGDex, CCGMel, CCGRef and CCGTad) were weighed and dissolved in 1 mL of distilled water, resulting in a solution of 1.33 mg of active substance/mL. Finally, control solutions of the active substances were prepared, so as to obtain the same concentration.

**Figure 9:**
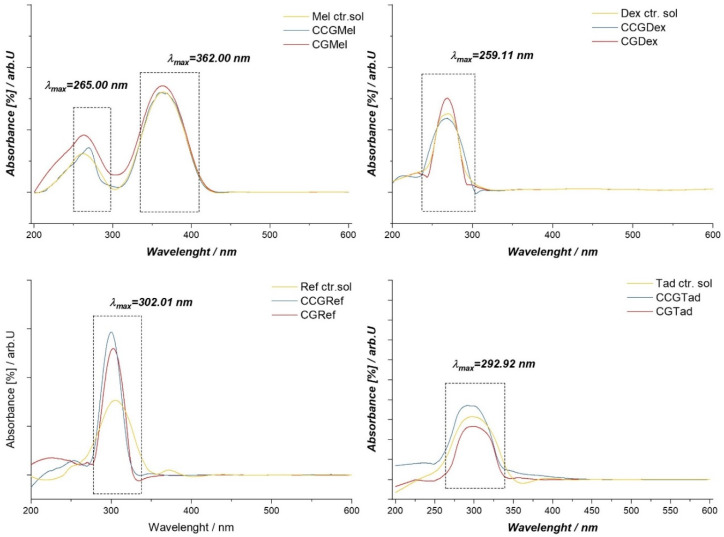
UV-VIS spectrum for control solutions of anti-inflammatory drugs and membranes in which these drugs were entrapped, both in type A and type B membranes.

In the case of membranes in which dex, CGDex, and CCGDex were incorporated, the UV-VIS spectrum had a maximum absorption at 259.01 nm (Figure 9). When the spectra of the samples were compared with those of the dexamethasone solution, as a control sample, the same maximum absorption was seen at 259.01 nm, as in the case of the membranes analyzed. In conclusion, we can say that the active substance was present in the membrane matrix and did not interact with it because the maximum at 258.01 nm would have been absent or shifted in the UV-VIS spectrum of the membranes.

For the Tad control solution, the UV-VIS spectrum had a maximum absorption at 292.92 nm, which was the same maximum observed in the case of membranes with active substances. For membranes in which Ref. had been incorporated, the UV-VIS spectrum had a maximum absorption at 302.01 nm. This maximum could also be found in the control solution spectrum, indicating that the active substance was intact in the CGRef and CCGRef membranes matrices.

The UV-VIS spectrum for Mel as a control solution indicated the presence of two absorption maxima, one at 362.00 nm and the other at 265.00 nm. Thus, comparing the spectra of CGMel and CCGMel membranes with those of the control, these two maxima could be found, which indicated the presence of the active substance in the membrane matrices.

All membranes contained active substances and these substances could be said not to interact with membrane components.

## 4. Conclusions

Although carrageenan, as a carrier for different active substances, has been used in different drug delivery systems over the past years, this study highlights a novel, simple, cheap and easily scalable method for production of thin polysaccharide membranes that can be used in a drug delivery system.

Following the study, two types of membranes were obtained in which four different anti-inflammatory drugs were incorporated. The IR spectra of the analyzed membranes show peaks that are characteristic of the functional groups of the active substances, and peaks characteristic of the membrane base. The FTIR study indicated that all the active substances were present intact within the membranes and no interactions between the active substance and the membrane matrices were highlighted, neither with the biopolymer nor with the plasticizer, glycerol. UV-VIS analysis determined the presence of active substances in the studied membranes. The TG analysis indicated good thermal stability in the temperature range of 37–100 °C for both membranes and entrapped active substances. The thermal analysis indicated the presence of the active substances in intact form. Their ability to “stick” to the skin could be an excellent property for membranes and patches intended for transdermal drug delivery. The development of stable anti-inflammatory drug membranes, as an efficient means of transdermal administration, may deliver the drug for an extended period of time, increase local soft tissue and joint concentrations, while avoiding the hepatic first–pass metabolism and reducing the gastrointestinal side effects associated with oral administration. Even if the synthesis of these polysaccharide membranes, doped with active substances, is a simple and efficient one, in order for these membranes to be used in the drug delivery system, an additional study is required regarding their effectiveness in releasing the active substance, either in vitro or in vivo, their mechanical resistance, as well as “packaging” and storage conditions, their shelf life, etc…

## Figures and Tables

**Table 1:** Type A and type B membranes with different anti-inflammatory drugs and their appearance.

Type A Membranes			Type B Membranes		
Abrev.	Membrane Appearance	Detail	Abrev.	Membrane Appearance	Detail
***CGRef***	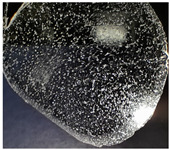	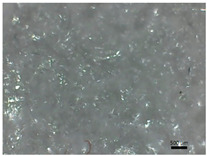	***CCGRef***	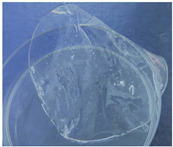	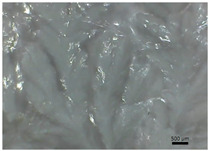
***CGTad***	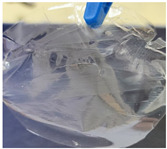	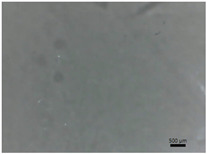	***CCGTad***	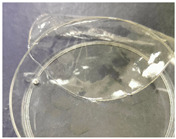	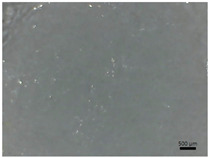
***CGMel***	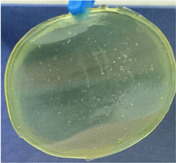	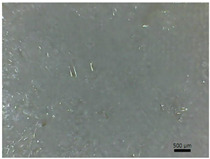	***CCGMel***	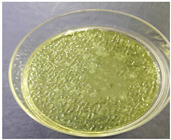	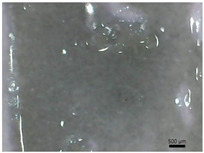
***CGDex***	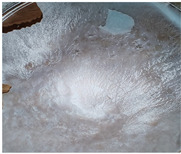	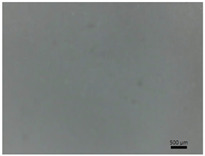	***CCGDex***	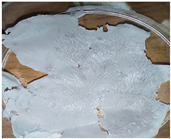	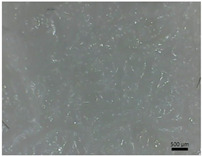

**Table 2:** Wavenumber values of most relevant peaks found in FT-IR spectra of analyzed membranes.

	*CG*	*CG Dex*	*CGRef*	*CGTad*	*CGMel*
	*Measured wavenumber of most relevant peaks (* *cm^−1^)*				
***O-H***	3315.63	3255.84	3275.13	3325.28	3398.57
***C-H***	2937.59	2926.01	2935.66	2935.66	2879.72
	2885.51	2860.43	2887.44	2887.44	
***C=O***	1647.21	1691.57	1674.21	1649.14	1647.21
***N-H deformation vibrations***		1579.70 A	1546.91	1568.13	1507.84
***CH_3_ and CH_2_ deformation vibration***	1419.61 B		1450.47	1452,40	1462.04 A
	1367.53 B	1390.68 B	1379.10 A	1394.53 B	1338.04 C
***C-N stretch vibration***	1220.94 A	1240.23 C	1228.66	1242.16	1240.23
***C-H sym. deformation vibration***	1161.15	1155.36 D	1155.36	1161.15	1141.86
***-H_2_C=CH- (from Ar)-OH stretching***					1103.28
***C-O stretching, linkage of 3,6-anhydro-D-galactose***	1031.46 918.63	1037.70 920.05	1033.85 920.05	1033.85 920.05	1064.71 956.69
***C-O-SO_3_ of D-galactose-4, sulfate***	844.82	842.89	842.89	844.82	840.96
***C-Cl stretching***			700.16		

Legend: A—Stretch vibrations C=C, 2,5-cyclohexadien-1-one ring; B—Stretch vibrations CH_3_, 2,5-cyclohexadien-1-one ring; C—Stretch vibrations P=O; D—Stretch vibrations C-F; A—S=O stretching vibration; B—Bending of O-H group; A, B—C=C stretch aromatic; C—S=O stretch from sulfone group; A—=C-H and ring C=C structural vibration.

**Table 3:** Wavenumber values of most relevant peaks found in FT-IR spectra of type B membranes.

	*CCG*	*CCG Dex*	*CCGRef*	*CCGTad*	*CCGMel*
	*Measured wavenumber of most relevant peaks (* *cm^−1^)*				
***O-H***	3296.35	3246.20	3246.20	3280.92	3360.00
***C-H***	2935.66	2937.59	2933.73	2933.73	2872.01
	2881.65	2883.58	2879.72	2881.65	
***C=O***	1649.14	1666.50	1649.14	1647.21	1627.92
***N-H deformation vibrations***		1579.70 A	1579.70	1570.06	1514.12
***CH_3_ and CH_2_ deformation vibration***	1415.75		1452.40	1452.40	1456.26 A
	1328.95 B	1390.68 B	1375.25 A	1394.53 B 1395.82 B	1334.74 C
***C-N stretch vibration***	1219.01 A	1244.09 C	1249.87	1284.16 1247.94	1240.23
***C-H sym. deformation vibration***	1159.22 1107.14	1111.00 D	1134.14	1105.21	1190.08
***-H_2_C=CH- (from Ar)-OH stretching***					1109.07
***C-O stretching, linkage of 3,6-anhydro-D-galactose***	1031.92 995.27 920.05	1039.63 987.55 918.12	1031.92 925.83	1033.85 921.97	1056.99 923.90
***C-O-SO3 of D-galactose-4, sulfate***	848.68	844.82	839.03	848.68	850.61
***C-Cl stretching***			669.30		

**Legend:** A—Stretch vibrations C=C, 2,5-cyclohexadien-1-one ring; B—Stretch vibrations CH_3_, 2,5-cyclohexadien-1-one ring; C— Stretch vibrations P=O; D—Stretch vibrations C-F; A—S=O stretching vibration; B—Bending of O-H group; A, B—C=C stretch aromatic; C—S=O stretch from sulfone group; A—=C-H and ring C=C structural vibration.

## Data Availability

Not applicable.

## References

[1] Lafargue D., Lourdin D., Doublier J.L. (2007). Film-forming properties of a modified starch/κ-carrageenan mixture in relation to its rheological behavior. Carbohydr. Polym..

[2] Vieira M.G.A., Da Silva M.A., Dos Santos L.O., Beppu M.M. (2011). Natural-based plasticizers and biopolymer films: A review. Eur. Polym. J..

[3] Saikia C., Gogoi P. (2015). Chitosan: A Promising Biopolymer in Drug Delivery Applications. J. Mol. Genet. Med..

[4] Oyarzun-Ampuero F., Brea J., Loza M.I., Torres D., Alonso M.J. (2009). Chitosan–hyaluronic acid nanoparticles loaded with heparin for the treatment of asthma. Int. J. Pharm..

[5] Rao N., Rho J.G., Um W., Ek P.K., Nguyen V.Q., Oh B.H., Kim W., Park J.H. (2020). Hyaluronic Acid Nanoparticles as Nanomedicine for Treatment of Inflammatory Diseases. Pharmaceutics.

[6] Ahmadian E., Dizaj S.M., Eftekhari A., Dalir E., Vahedi P., Hasanzadeh A., Samiei M. (2020). The Potential Applications of Hyaluronic Acid Hydrogels in Biomedicine. Drug Res..

[7] Alibadi S.S., Mohammadifar M.A., Hosseini H., Mohammadi A., Ghasemlou M., Hosseini S.M., Haghshenas M., Khaksar R. (2014). Characterization of nanobiocomposite kappa-carrageenan film with Zataria multiflora essential oil and nanoclay. Int. J. Biol. Macromol..

[8] El-Aassar M.R., El Fawal G.F., Kamoun E.A., Fouda M.M.G. (2015). Controlled drug release from cross-linked κ-carrageenan /hyaluronic acid membranes. Int. J. Biol. Macromol..

[9] Goff H.D., Guo Q. (2020). Chapter 1. The Role of Hydrocolloids in the Development of Food Structure. Handbook of Food Structure Development.

[10] Campo V.L., Kawano D.F., Da Silva D.B., Carvalho I. (2009). Carrageenans: Biological properties, chemical modifications and structural analysis—A review. Carbohydr. Polym..

[11] Andersonn N.S., Dolan T.C.S., Lawson C.J., Penman A., Rees D.A. (1968). Carrageenans: Part V. The masked repeating structures of λ- and μ-carrageenans. Carbohydr. Res..

[12] Masakuni T. (2015). The Principle of Polysaccharide Gels. Adv. Biosci. Biotechnol..

[13] Sutrisni N.N.W., Soewandhi S.N., Adnyana I.K., Sasongko L.D.N. (2019). Acute and Subchronic (28-day) Oral Toxicity Studies on the Film Formulation of k-Carrageenan and Konjac Glucomannan for Soft Capsule Application. Sci. Pharm..

[14] Ariffin S.H.Z., Yeen W.W., Abidin I.Z.Z., Wahab R.M.A., Ariffin Z.Z., Senafi S. (2014). Cytotoxicity effect of degraded and undegraded kappa and iota carrageenan in human intestine and liver cell lines. BMC Complement. Altern. Med..

[15] Rainsford K.D. (2007). Chapter 1. Anti-Inflammatory Drugs in the 21-st Century. Inflammation in the Pathogenesis of Chronic Diseases.

[16] Flower R.J. (1974). Drugs which inhibit prostaglandin biosynthesis. Pharmacol. Rev..

[17] Vane J.R., Botting R.M. (1998). Anti-inflammatory drugs and their mechanism of action. Agents Actions.

[18] Gan T.J. (2010). Diclofenac: An update on its mechanism of action and safety profile. Curr. Med Res. Opin..

[19] Uwah T., Akpabio E., Effiong D., Akpabio A., Godwin J. (2018). Preliminary investigations into the physicochemical and compaction characteristics of modified starch of discorea data using diclofenac sodium tablet. Int. J. Pharm. Pharm. Sci..

[20] Birmingham B., Buvanendran A. (2013). Nonsteroidal anti-inflammatory drugs, acetaminophen, and COX-2 inhibitors. Practical Management of Pain.

[21] Evanson N.K., Enna S.J., Bylund D.B. (2007). Meloxicam. xPharm: The Comprehensive Pharmacology Reference.

[22] Hanna M., Moon J.Y. (2019). A review of dexketoprofen trometamol in acute pain. Curr. Med Res. Opin..

[23] Chandrasekharan N. (2007). Dexketoprofen. xPharm: The Comprehensive Pharmacology Reference.

[24] El-Malla S. (2014). Spectroscopic Methods for Determination of Dexketoprofen Trometamol and Tramadol HCl. Inventi Impact Pharm Anal Qual. Assur..

[25] Ahmed M.H., Hassan A. (2020). Dexamethasone for the Treatment of Coronavirus Disease (COVID-19): A Review. SN Compr. Clin. Med..

[26] Wang Q., Liu X., Su M., Shi Z., Sun H. (2014). Study on the interaction characteristics of dexamethasone sodium phosphate with bovine serum albumin by spectroscopic technique. New J. Chem..

[27] Al-Owaidi M.F., Alkhafaji S.L., Mahood A.M. (2021). Quantitative determination of dexamethasone sodium phosphate in bulk and pharmaceuticals at suitable pH values using the spectrophotometric method. J. Adv. Pharm. Technol. Res..

[28] Bajas D., Vlase G., Mateescu M., Grad O.A., Bunoiu M., Vlase T., Avram C. (2021). Formulation and characterization of alginate-based membranes for the potential transdermal delivery of methotrexate. Polymers.

[29] Marioane C.A., Bunoiu M., Mateescu M., Sfîrloagă P., Vlase G., Vlase T. (2021). Preliminary Study for the Preparation of Transmucosal or Transdermal Patches with Acyclovir and Lidocaine. Polymers.

[30] Rukmanikrishnan B., Ramalingam S., Kim S.S., Lee J. (2021). Rheological and anti-microbial study of silica and silver nanoparticles-reinforced k-carrageenan/hydroxyethyl cellulose composites for food packaging applications. Cellulose.

[31] Liew J., Loh K.S., Ahmad A., Lim K.L., Wan Daud W. (2017). Synthesis and characterization of modified κ-carrageenan for enhanced proton conductivity as polymer electrolyte membrane. PLoS ONE.

